# The association of hypoglycemia with outcome of critically ill children in relation to nutritional and blood glucose control strategies

**DOI:** 10.1186/s13054-023-04514-6

**Published:** 2023-06-26

**Authors:** Jan Gunst, Astrid De Bruyn, An Jacobs, Lies Langouche, Inge Derese, Karolijn Dulfer, Fabian Güiza, Gonzalo Garcia Guerra, Pieter J. Wouters, Koen F. Joosten, Sascha C. Verbruggen, Ilse Vanhorebeek, Greet Van den Berghe

**Affiliations:** 1 Clinical Division and Laboratory of Intensive Care Medicine, Department of Cellular and Molecular Medicine, KU Leuven, B-3000 Leuven, Belgium; 2 Intensive Care Unit, Department of Pediatrics and Pediatric Surgery, Erasmus Medical Center, Sophia Children’s Hospital, Rotterdam, The Netherlands; 3 Intensive Care Unit, Department of Pediatrics, University of Alberta, Stollery Children’s Hospital, Edmonton, Canada; 4 Present Address: Pediatric Intensive Care Unit, Department of Pediatrics, University of Calgary, Alberta Children’s Hospital, Calgary, Canada

**Keywords:** Hypoglycemia, Intensive care, Parenteral nutrition, Neurodevelopment, Insulin, Tight glucose control

## Abstract

**Background:**

Withholding parenteral nutrition (PN) until one week after PICU admission facilitated recovery from critical illness and protected against emotional and behavioral problems 4 years later. However, the intervention increased the risk of hypoglycemia, which may have counteracted part of the benefit. Previously, hypoglycemia occurring under tight glucose control in critically ill children receiving early PN did not associate with long-term harm. We investigated whether hypoglycemia in PICU differentially associates with outcome in the context of withholding early PN, and whether any potential association with outcome may depend on the applied glucose control protocol.

**Methods:**

In this secondary analysis of the multicenter PEPaNIC RCT, we studied whether hypoglycemia in PICU associated with mortality (*N* = 1440) and 4-years neurodevelopmental outcome (*N* = 674) through univariable comparison and multivariable regression analyses adjusting for potential confounders. In patients with available blood samples (*N* = 556), multivariable models were additionally adjusted for baseline serum NSE and S100B concentrations as biomarkers of neuronal, respectively, astrocytic damage. To study whether an association of hypoglycemia with outcome may be affected by the nutritional strategy or center-specific glucose control protocol, we further adjusted the models for the interaction between hypoglycemia and the randomized nutritional strategy, respectively, treatment center. In sensitivity analyses, we studied whether any association with outcome was different in patients with iatrogenic or spontaneous/recurrent hypoglycemia.

**Results:**

Hypoglycemia univariably associated with higher mortality in PICU, at 90 days and 4 years after randomization, but not when adjusted for risk factors. After 4 years, critically ill children with hypoglycemia scored significantly worse for certain parent/caregiver-reported executive functions (working memory, planning and organization, metacognition) than patients without hypoglycemia, also when adjusted for risk factors including baseline NSE and S100B. Further adjustment for the interaction of hypoglycemia with the randomized intervention or treatment center revealed a potential interaction, whereby tight glucose control and withholding early PN may be protective. Impaired executive functions were most pronounced in patients with spontaneous or recurrent hypoglycemia.

**Conclusion:**

Critically ill children exposed to hypoglycemia in PICU were at higher risk of impaired executive functions after 4 years, especially in cases of spontaneous/recurrent hypoglycemia.

## Background

Hypoglycemia is associated with increased mortality in critically ill children [1] and with intracranial hemorrhage and infarction, basal ganglia and thalamic abnormalities, as well as cortical lesions in non-critically ill neonates [2]. However, it remains debated whether brief hypoglycemia occurring in the context of critical illness by itself causes harm, since hypoglycemia more frequently occurs in patients with higher illness severity, which introduces potential bias [3]. Indeed, prolonged underfeeding and liver failure, more frequently occurring in sicker patients, predispose to hypoglycemia [3]. Likewise, a longer duration of stay in the pediatric intensive care unit (PICU), also indicating higher illness severity, prolongs the exposure to glucose control strategies and hence increases the odds of developing a hypoglycemic event.

Besides hypoglycemia, also hyperglycemia and high glucose variability have been associated with poor outcome of critically ill children [1, 4, 5]. Hyperglycemia associated with neuronal damage in the hippocampus and frontal cortex of adult patients who died in the intensive care unit [6]. Some observational studies of critically ill children have attributed an even higher risk of poor outcome to hyperglycemia and large glucose variability than to hypoglycemia alone [1, 5]. Moreover, a randomized controlled trial (RCT) in critically ill children (*N* = 700) showed that prevention of hyperglycemia by tight glucose control not only reduced short-term morbidity and mortality, but also slightly improved long-term neurodevelopmental outcome assessed 4 years later, despite a 25-fold elevated risk of hypoglycemia [7, 8]. In this RCT, patients who developed hypoglycemia already had higher baseline serum concentrations of neuron-specific enolase (NSE) and S100 calcium binding protein B (S100B) as compared with patients without hypoglycemia [9]. Moreover, these biomarkers of neuronal, respectively, astrocytic damage did not increase after the hypoglycemic event, and patients who developed hypoglycemia under tight glucose control did not have worse 4-year neurodevelopmental outcome as compared with propensity score-matched patients without hypoglycemia [8, 9]. Altogether, this evidence suggests that a brief episode of predominantly iatrogenic hypoglycemia is less harmful for the developing brain than prolonged hyperglycemia. However, the association of hypoglycemia with outcome may be context-dependent. Indeed, in the glucose control RCT, patients received early parenteral nutrition (PN) which increases the severity of stress hyperglycemia. This nutritional strategy was subsequently shown to be harmful in the PEPaNIC RCT (*N* = 1440) [10]. The PEPaNIC RCT revealed that withholding early PN until one week after PICU admission (Late-PN) was superior to early supplementation of insufficient enteral nutrition by parenteral nutrition (Early-PN) [10]. Late-PN enhanced recovery from critical illness and protected against emotional and behavioral problems 4 years after randomization [10, 11]. At the same time, the intervention almost doubled the incidence of hypoglycemia in the first PICU week, which may theoretically have counteracted part of the benefit [10]. Although hypoglycemia did not independently associate with short- or long-term harm in the glucose control study [8, 9], it is not clear whether these findings also apply to patients who do not receive early PN and to patients who do not receive tight glucose control. In the PEPaNIC RCT, the majority of hypoglycemic events occurred in patients not receiving early PN, and the glucose control protocol varied per center, going from tight over intermediate to liberal glucose control [10]. Different feeding and glucose control strategies have a different risk of iatrogenic hypoglycemia and may variably affect generation of ketone bodies that are vital alternative substrates for the brain during hypoglycemia [12–14].

In this secondary analysis of the PEPaNIC RCT, we investigated whether hypoglycemia during PICU stay independently associates with impaired short- and long-term mortality and with 4-years neurodevelopmental outcome of critically ill children, and whether such association—if any—is affected by the randomized nutritional strategy and the glucose control protocol. In addition, we studied whether any association between hypoglycemia and outcome is dependent on whether hypoglycemia was a single iatrogenic event or had occurred recurrently/spontaneously.

## Methods

### Study design

This study is a secondary analysis of the multicenter PEPaNIC RCT (clinicaltrials.gov NCT01536275), in which 1440 critically ill children admitted to participating PICUs (University Hospitals Leuven, Belgium; Erasmus Medical Centre–Sophia Children’s Hospital, Rotterdam, the Netherlands; and Stollery Children’s Hospital, Edmonton, AB, Canada) were randomized to receive early PN when enteral nutrition was contraindicated or insufficient to meet the caloric target (Early-PN), or to withhold PN until one week after PICU admission (Late-PN), hereby accepting a relative macronutrient deficit in the first week in PICU [10]. Participating centers had a center-specific glucose control protocol. In Leuven, continuous intravenous insulin was used to target 50–80 mg/dL (2.8–4.4 mmol/L) in infants and 70–100 mg/dL (3.9–5.6 mmol/L) in children older than one year. Blood glucose and potassium concentrations were measured every 1 to 4 h on arterial blood with use of a blood gas analyzer (ABL Radiometer, Copenhagen, Denmark). In Rotterdam, the blood glucose target was 72–145 mg/dL (4.0–8.0 mmol/L), except for patients admitted with traumatic brain injury, for whom the target was 108–145 mg/dL (6.0–8.0 mmol/L). Blood glucose and potassium concentrations were measured every 1 to 3 h on arterial or capillary blood using a blood gas analyzer (ABL 625, Radiometer, Copenhagen, Denmark). In Edmonton, insulin was only initiated when blood glucose concentrations exceeded 180 mg/dL (10.0 mmol/L), without a specific lower glucose boundary for patients requiring insulin infusion [10, 15]. The study was performed in accordance with the 1964 Declaration of Helsinki and later amendments. The protocol was approved by the institutional or national ethical review boards of participating centers. The parents or legal guardians provided written informed consent. The impact of the intervention on acute clinical outcome and 4-years neurodevelopmental outcome has been published previously [10, 11].

### Outcomes

We investigated the association between the occurrence of hypoglycemia (< 40 mg/dL [< 2.2 mmol/L]) during PICU stay with mortality in PICU, at 90 days and 4 years after randomization, and with neurodevelopmental outcomes after 4 years. Neurodevelopmental outcomes were scored by clinical tests and by validated, internationally recognized questionnaires, and included relevant problems in clinical neurological development, executive functions, behavioral and emotional development, and general intellectual functioning [11]. During clinical neurological evaluation, 8 domains were scored as normal (0) or abnormal (1), yielding a total score from 0 to 8 [11]. The assessed domains included a gross evaluation of interaction and language skills, gross motor function, involuntary movements, reflexes, coordination and balance, fine motor function, cranial nerves, and special senses (sensory, visual, and auditory functions). To evaluate executive functioning, parents and caregivers were asked to complete the Behavior Rating Inventory of Executive Function (BRIEF) questionnaires on executive functioning of their child, which allowed analysis of inhibitory control, cognitive flexibility, emotional control, working memory, planning and organization, metacognition, and total executive functioning [16, 17]. To asses behavioral and emotional problems, Child Behavior Checklist (CBCL) questionnaires were used to analyze internalizing, externalizing, and total behavioral and emotional problems [18, 19]. The scores for executive functions and for behavioral and emotional problems were reported as T scores, with a higher score representing more problems. To score general intellectual ability, the age-appropriate version of the Wechsler Intelligence Quotients (IQ) scale was used [20–22]. Total IQ was reported as T score, with higher scores representing a better general intellectual ability.

### Biochemical analyses

Serum NSE and S100B concentrations were measured by commercial enzyme-linked immunosorbent assay (ELISA) kits (Human Enolase 2/Neuron-specific Enolase Quantikine ELISA, R&D Systems, Abingdon, UK; S100B Human ELISA, BioVendor GmbH, Heidelberg, Germany) on stored samples as per manufacturer’s recommendations. We analyzed blood samples taken upon PICU admission (or on the first morning in PICU, if no admission sample was available). After harvesting, blood samples were stored at − 80 °C until further analysis.

### Statistical analyses

Since a failure to complete the neurodevelopmental tests can be expected for patients with poor neurocognitive function, we performed multiple data imputation by chained equations to address partial responses on the 4-years neurodevelopmental tests, with use of all available data for each participant [11, 23]. To avoid bias and instability of the model, imputation was only performed for outcomes with maximum 30% of missing data. The number of imputation models was set at 31 to avoid loss of statistical power [11, 23].

First, the association between hypoglycemia in PICU and mortality and long-term neurodevelopmental outcomes was investigated by univariable comparison and by multivariable regression analyses adjusted for baseline risk factors. Baseline risk factors were age, sex, treatment center, randomization to Early-PN or Late-PN, severity of illness upon PICU admission (pediatric index of mortality 3 [PIM3] score), risk of malnutrition (medium or high risk according to the Screening Tool for Risk On Nutritional Status and Growth [STRONGkids] score), admission diagnostic category, history of malignancy, diabetes, and a predefined syndrome. Multivariable models for the 4-year neurodevelopmental outcomes were additionally adjusted for race, geographic origin, language, hand preference, the educational and occupational level of the parents, and parental smoking before PICU admission. Second, for those outcomes revealing a significant independent association with hypoglycemia, we repeated multivariable regression analyses further adjusting for length of PICU stay, mean morning blood glucose, and glucose variability (standard deviation of all glucose measurements in PICU) as potential confounders. For those outcomes with a persistent independent association with hypoglycemia, we studied whether the association may be affected by the randomized nutritional strategy or the center-specific glucose control protocol. For this purpose, the multivariable models were additionally adjusted for the interaction between the randomized intervention and the occurrence of hypoglycemia in PICU, respectively, the interaction between the treatment center and the occurrence of hypoglycemia.

Since our group had previously observed elevated baseline NSE and S100B in patients who subsequently developed hypoglycemia as compared with those who did not, we repeated the multivariable regression analyses described above, in the subset of patients with available blood sample, further adjusting for the upon-admission (or first morning) NSE and S100B concentrations as biomarkers of baseline neuronal and astrocytic damage.

For those outcomes revealing a consistent significant independent association with hypoglycemia, sensitivity analyses were performed to investigate whether an episode of iatrogenic hypoglycemia differentially associates with outcome as compared with spontaneous or recurrent hypoglycemia, first in univariable comparison and thereafter in multivariable regression analysis adjusting for baseline risk factors, mean morning blood glucose, glucose variability, and length of PICU stay. Iatrogenic hypoglycemia was defined as hypoglycemia developing under insulin treatment or within 2 h after stopping insulin.

Data are presented as frequencies and percentages, mean (standard deviation), or median (25th–75th percentile), as appropriate. We performed Chi-square and Fisher’s exact test, Wilcoxon rank-sum or Kruskal–Wallis test to study univariate comparisons, and multivariable logistic and linear regression analyses to study multivariable comparisons, as appropriate. Two-sided *p*-values < 0.05 were considered statistically significant, without correction for multiple comparisons. To study interactions, interaction *p*-values < 0.15 were considered statistically significant. Statistical analyses were performed with JMP© Pro version 17.0.0 (JMP, Marlow, Buckinghamshire, UK).

## Results

### Association between hypoglycemia and short- and long-term mortality

The association between hypoglycemia and subsequent mortality was assessed in the total cohort of patients included in the PEPaNIC trial (*n* = 1440). Hypoglycemia in PICU developed in 121 patients (8.4%). Baseline characteristics, glucose metrics, and outcome of patients with and without hypoglycemia are shown in Table 1. Patients with hypoglycemia were significantly younger (*P* < 0.0001), more often treated in Leuven (*P* < 0.0001), more frequently randomized to Late-PN (*P* = 0.046) and categorized in different diagnostic groups (*P* < 0.0001), with a higher baseline illness severity score (PIM3 score; *P* < 0.0001) than patients without hypoglycemia. Mean morning blood glucose was significantly lower, glucose variability was larger, and PICU stay was significantly longer (all *P* < 0.0001). Patients with hypoglycemia had a higher mortality in PICU (*P* = 0.003), at 90 days (*P* = 0.0005), and 4 years after randomization (*P* = 0.005). In multivariable analyses adjusted for baseline risk factors, the occurrence of hypoglycemia did not independently associate with mortality (all *P* > 0.3; Table 2).

**Table 1:** Baseline and clinical characteristics, and outcome of the total and neurodevelopmental follow-up cohort

	Total PEPaNIC study cohort (*N* = 1440)			Neurodevelopmental follow-up cohort (*N* = 674)			Neurodevelopmental follow-up cohort with available baseline NSE/S100B (*N* = 556)		
	Patients without hypoglycemia in PICU (*N* = 1319)	Patients with hypoglycemia in PICU (*N* = 121)	*P*	Patients without hypoglycemia in PICU (*N* = 614)	Patients with hypoglycemia in PICU (*N* = 60)	*P*	Patients without hypoglycemia in PICU (*N* = 500)	Patients with hypoglycemia in PICU (*N* = 56)	*P*
*Baseline characteristics*									
Age (years)	1.8 (0.3–7.2)	0.2 (0.02–0.5)	< 0.0001	1.5 (0.2–5.2)	0.1 (0.02–0.5)	< 0.0001	2.0 (0.4–6.0)	0.1 (0.02–0.5)	< 0.0001
Male sex	765 (58.0%)	65 (53.7%)	0.39	353 (57.5%)	35 (58.3%)	0.90	280 (56.0%)	34 (60.7%)	0.57
Treatment center			< 0.0001			< 0.0001			0.0003
Leuven	656 (49.7%)	94 (77.7%)		346 (56.4%)	53 (88.3%)		344 (68.8%)	51 (91.1%)	
Rotterdam	569 (43.1%)	24 (19.8%)		268 (43.6%)	7 (11.7%)		156 (31.2%)	5 (8.9%)	
Edmonton	94 (7.1%)	3 (2.5%)							
Randomization to Late-PN	646 (49.0%)	71 (58.7%)	0.046	313 (51.0%)	40 (66.7%)	0.022	254 (50.8%)	40 (71.4%)	0.004
PIM3 score	− 3.6 (− 4.4 to − 2.5)	− 2.5 (− 3.6 to − 1.3)	< 0.0001	− 3.9 (− 4.5 to − 2.8)	− 3.1 (− 3.6 to − 1.8)	< 0.0001	− 3.9 (− 4.4 to − 2.8)	− 3.1 (− 3.6 to − 1.8)	< 0.0001
PIM3 probability of death	2.7% (1.2–7.6%)	7.8% (2.6–21.1%)	< 0.0001	2.0% (1.1–5.9%)	4.3% (2.6–14.5%)	< 0.0001	2.1% (1.2–5.7%)	4.4% (2.7–14.5%)	< 0.0001
STRONGkids risk level			0.28			0.89			0.99
Medium	1176 (89.2%)	112 (92.6%)		549 (89.4%)	54 (90.0%)		457 (91.4%)	52 (92.9%)	
High	143 (10.8%)	9 (7.4%)		65 (10.6%)	6 (10.0%)		43 (8.6%)	4 (7.1%)	
Diagnostic category			< 0.0001			< 0.0001			0.0002
Surgical–cardiac	475 (36.0%)	72 (59.5%)		250 (40.7%)	38 (63.3%)		245 (49.0%)	37 (66.1%)	
Surgical–other	213 (16.1%)	9 (7.4%)		127 (20.7%)	3 (5.0%)		69 (13.8%)	1 (1.8%)	
Neurosurgery–neurology	215 (16.3%)	4 (3.3%)		97 (15.8%)	3 (5.0%)		86 (17.2%)	3 (5.4%)	
Trauma–burn	63 (4.8%)	1 (0.8%)		22 (3.6%)	0 (0.0%)		22 (4.4%)	0 (0.0%)	
Transplantation–hematology–oncology	38 (2.9%)	1 (0.8%)		16 (2.6%)	0 (0.0%)		12 (2.4%)	0 (0.0%)	
Medical–other	315 (23.9%)	34 (28.1%)		102 (16.7%)	16 (26.7%)		66 (13.2%)	15 (26.8%)	
History of malignancy	81 (6.1%)	3 (2.5%)	0.11	37 (6.0%)	1 (1.7%)	0.24	34 (6.8%)	1 (1.8%)	0.24
Diabetes	2 (0.2%)	1 (0.1%)	0.23	0 (0.0%)	0 (0.0%)	–	0 (0.0%)	0 (0.0%)	–
Predefined syndrome	220 (16.7%)	21 (17.4%)	0.80	55 (9.0%)	6 (10.0%)	0.81	51 (10.2%)	6 (10.7%)	0.82
*Demographics*									
Known non-white race	–	–		51 (8.3%)	2 (3.3%)	0.21	36 (7.2%)	2 (3.6%)	0.41
Known non-European origin	–	–		110 (17.9%)	9 (15.0%)	0.72	85 (17.0%)	9 (16.1%)	0.99
Known not exclusive Dutch or English language	–	–		144 (23.4%)	12 (20.0%)	0.63	119 (23.8%)	11 (19.6%)	0.62
Socioeconomic status^1^									
Parental educational level						0.33			0.22
Level 1	–	–		24 (3.9%)	6 (10.0%)		18 (3.6%)	6 (10.7%)	
Level 1.5	–	–		47 (7.7%)	4 (6.7%)		39 (7.8%)	4 (7.1%)	
Level 2	–	–		141 (23.0%)	16 (26.7%)		117 (23.4%)	14 (25.0%)	
Level 2.5	–	–		105 (17.1%)	11 (18.3%)		82 (16.4%)	11 (19.6%)	
Level 3	–	–		170 (27.7%)	13 (21.7%)		145 (29.0%)	12 (21.4%)	
Level unknown	–	–		127 (20.7%)	10 (16.7%)		99 (19.8%)	9 (16.1%)	
Parental occupational level						0.075			0.065
Level 1	–	–		4 (0.7%)	2 (3.3%)		4 (0.8%)	2 (3.6%)	
Level 1.5	–	–		55 (9.0%)	7 (11.7%)		46 (9.2%)	7 (12.5%)	
Level 2	–	–		92 (15.0%)	16 (26.7%)		85 (17.0%)	16 (28.6%)	
Level 2.5	–	–		63 (10.3%)	4 (6.7%)		52 (10.4%)	4 (7.1%)	
Level 3	–	–		108 (17.6%)	9 (15.0%)		93 (18.6%)	9 (16.1%)	
Level 3.5	–	–		51 (8.3%)	1 (1.7%)		40 (8.0%)	0 (0.0%)	
Level 4	–	–		93 (15.1%)	8 (13.3%)		76 (15.0%)	8 (14.3%)	
Level unknown	–	–		148 (24.1%)	13 (21.7%)		105 (21.0%)	10 (17.9%)	
Known parental smoking between birth and PICU admission	–	–		172 (28.0%)	14 (23.3%)	0.55	130 (26.0%)	13 (23.2%)	0.75
Left-hand preference	–	–		86 (14.0%)	9 (15.0%)	0.85	70 (14.0%)	9 (16.1%)	0.69
*Baseline biomarkers*									
NSE (ng/mL)	–	–		–	–		9 (7–13)	12 (8–17)	0.001
S100B (pg/mL)	–	–		–	–		29 (11–55)	38 (19–63)	0.078
*In PICU characteristics*^2^									
Mean morning blood glucose (mg/dL)	107 (93–125)	91 (84–102)	< 0.0001	104 (91–121)	89 (82–99)	< 0.0001	104 (90–122)	89 (82–98)	< 0.0001
SD of all glucose measurements in PICU (mg/dL)	27 (18–43)	35 (27–48)	< 0.0001	26 (17–41)	32 (25–42)	0.005	27 (19–42)	31 (25–42)	0.055
Length of PICU stay (days)	3 (2–7)	8 (4–20)	< 0.0001	3 (2–6)	8 (4–19)	< 0.0001	3 (2–6)	8 (5–19)	< 0.0001
*Mortality*									
in PICU	55 (4.2%)	13 (10.7%)	0.003	–	–		–	–	
at 90 days	70 (5.3%)	17 (14.0%)	0.0005	–	–		–	–	
at 4 years	116 (8.8%)	21 (17.4%)	0.005	–	–		–	–	
*Neurodevelopmental outcome after 4 years*^3^									
Clinical neurological evaluation score^4^, range 0–8	–	–		0.6 (1.2)	0.4 (1.0)	0.38	0.5 (1.2)	0.5 (1.0)	0.75
Executive functioning as reported by parents or caregivers, T-score^4^									
Inhibition	–	–		49.6 (11.1)	51.4 (12.6)	0.34	49.3 (11.0)	51.6 (12.8)	0.25
Flexibility	–	–		49.3 (10.2)	48.9 (9.6)	0.81	49.0 (9.9)	49.3 (9.7)	0.80
Emotional control	–	–		48.9 (9.8)	49.0 (11.2)	0.66	48.6 (9.7)	49.3 (11.5)	0.89
Working memory	–	–		51.4 (11.1)	55.7 (12.8)	0.015	51.3 (11.1)	56.3 (12.9)	0.006
Planning and organization	–	–		49.9 (10.2)	54.2 (12.8)	0.012	49.8 (10.4)	54.6 (12.9)	0.008
Metacognition index	–	–		50.1 (10.9)	54.0 (13.3)	0.046	50.0 (10.9)	54.5 (13.5)	0.025
Total score	–	–		49.5 (11.2)	52.4 (13.2)	0.15	49.3 (11.1)	52.8 (13.4)	0.082
Emotional and behavioral problems as reported by parents or caregivers, T-score^4^									
Internalizing problems	–	–		51.1 (10.7)	49.7 (11.6)	0.33	51.2 (10.6)	49.6 (11.9)	0.30
Externalizing problems	–	–		48.6 (10.0)	49.2 (10.0)	0.97	48.7 (9.7)	49.4 (10.1)	0.93
Total problems	–	–		50.1 (10.6)	49.1 (11.4)	0.34	50.3 (10.2)	49.3 (11.7)	0.36
Intelligence^5^									
Total IQ, range 45–155	–	–		93.3 (16.8)	94.1 (16.1)	0.79	93.6 (17.1)	93.7 (16.6)	0.93

Data are presented as median (25th–75th percentile), mean (SD), or n (%)

NSE: neuron-specific enolase

PICU: pediatric intensive care unit

PIM3 score: Pediatric Index of Mortality 3, with a higher score indicating a higher risk of mortality

S100B: S100 calcium binding protein B

STRONGkids: Screening Tool for Risk On Nutritional Status and Growth

^1^The education level is the mean of the paternal and maternal educational level, calculated on a 3-point based scale (1 is low, 2 is middle, and 3 is high). The occupational level is the mean of the paternal and maternal occupational level, which is calculated based on the International ISCO System 4-point scale for professions

^2^Mean morning blood glucose and SD of all glucose measurements are missing in 69 patients of the total cohort, and in 17, respectively, 11 patients without hypoglycemia of the neurodevelopmental follow-up cohort. Mean morning blood glucose is missing in 4 patients without hypoglycemia of the NSE/S100B cohort

^3^For executive functioning and emotional and behavioral problems reported by parents or caregivers, data from 19 (neurodevelopmental follow-up cohort), respectively, 17 (NSE/S100B cohort) patients without hypoglycemia are missing

^4^Higher scores reflect worse performance

^5^Higher scores reflect better performance

**Table 2:** Multivariable regression analyses investigating the independent association of hypoglycemia in PICU with outcome

	Patients with vs. patients without hypoglycemia	*P*	Patients with vs. without hypoglycemia; after further adjustment for baseline NSE/S100B	*P*
*Mortality*	Odds ratio (95% CI)		Odds ratio (95% CI)	
In PICU	1.20 (0.51–2.83)	0.69	–	
At 90 days	0.66 (0.31–1.43)	0.30	–	
At 4 years	0.71 (0.37–1.36)	0.31	–	
*Neurodevelopmental outcome after 4 years*^1^	β-Estimate (95% CI)		β-Estimate (95% CI)	
Clinical neurological evaluation score^2^, range 0–8	0.09 (− 0.06 to 0.23)	0.25	0.07 (− 0.09 to 0.22)	0.39
Executive functioning as reported by parents or caregivers, T-score^2^				
Inhibition	1.26 (− 0.32 to 2.84)	0.12	1.09 (− 0.59 to 2.77)	0.20
Flexibility	0.01 (− 1.41 to 1.44)	0.99	0.16 (− 1.31 to 1.64)	0.83
Emotional control	− 0.08 (− 1.52 to 1.35)	0.91	− 0.17 (− 1.70 to 1.36)	0.83
Working memory	2.17 (0.60 to 3.75)	0.007	2.36 (0.68 to 4.04)	0.006
Planning and organization	2.03 (0.53 to 3.53)	0.008	2.27 (0.65 to 3.89)	0.006
Metacognition index	1.85 (0.28 to 3.42)	0.021	2.08 (0.39 to 3.76)	0.016
Total score	1.36 (− 0.25 to 2.96)	0.10	1.52 (− 0.20 to 3.23)	0.084
Emotional and behavioral problems as reported by parents or caregivers, T-score^2^				
Internalizing problems	− 0.11 (− 1.60 to 1.37)	0.88	− 0.38 (− 1.95 to 1.19)	0.64
Externalizing problems	− 0.16 (− 1.54 to 1.22)	0.82	− 0.34 (− 1.80 to 1.11)	0.64
Total problems	− 0.14 (− 1.60 to 1.32)	0.85	− 0.31 (− 1.84 to 1.22)	0.69
Intelligence^3^				
Total IQ, range 45–155	0.24 (− 1.85 to 2.33)	0.82	0.52 (− 1.69 to 2.72)	0.65

Odds ratios for mortality outcomes are calculated with multivariable logistic regression analyses adjusted for age, sex, treatment center, randomization group, pediatric index of mortality (PIM3 score) at time of admission, Screening Tool for Risk on Nutritional Status and Growth (STRONGkids) risk level at time of admission, diagnostic category, history of malignancy, diabetes, and predefined syndrome. β-Estimates for neurodevelopmental outcomes are calculated with multivariable linear regression analyses performed on the 31 imputed datasets. These multivariable analyses are additionally adjusted for race, geographic origin, language, socioeconomic status (parental educational and occupational level), parental smoking before admission, left-hand preference, and (for the two columns on the right, as indicated) for upon-admission concentrations of NSE and S100B

^1^For executive functioning and emotional and behavioral problems reported by parents or caregivers, data from 19 (neurodevelopmental follow-up cohort), respectively, 17 (NSE/S100B cohort) patients without hypoglycemia are missing

^2^Higher scores reflect worse performance

^3^Higher scores reflect better performance

### Association between hypoglycemia and 4-year neurodevelopmental outcomes

Of the 1440 children included in the PEPaNIC RCT, 684 had detailed neurodevelopmental follow-up 4 years later [11]. We excluded all patients from Edmonton (*n* = 10), since none of the three patients developing hypoglycemia in this center had neurodevelopmental follow-up. Table 1 summarizes the baseline and clinical characteristics, as well as the outcomes for the study cohort of 674 patients. As in the total cohort, patients with hypoglycemia (*n* = 60) were significantly younger (*P* < 0.0001), more often treated in Leuven (*P* < 0.0001), more frequently randomized to Late-PN (*P* = 0.022), categorized in different diagnostic groups (*P* < 0.0001), and had a higher baseline illness severity (*P* < 0.0001) than patients without hypoglycemia (*n* = 614). Likewise, critically ill children with hypoglycemia had a lower mean morning blood glucose (*P* < 0.0001), larger glucose variability (*P* = 0.005), and a longer PICU stay (*P* < 0.0001). As compared with patients without hypoglycemia, patients with hypoglycemia had significantly worse scores for working memory (*P* = 0.015), planning and organization (*P* = 0.012), and metacognition (*P* = 0.046). Other neurodevelopmental outcomes were not significantly different. After adjustment for baseline risk factors, hypoglycemia remained independently associated with worse scores for working memory (*P* = 0.007), planning and organization (*P* = 0.008), and metacognition (*P* = 0.021) (Table 2). Additionally adjusting for mean morning blood glucose, glucose variability, and length of PICU stay did not change these findings (*P* = 0.015 for working memory, *P* = 0.026 for planning and organization, *P* = 0.049 for metacognition; Table 3). Further adjustment of the multivariable models for the potential interaction between hypoglycemia and the randomized intervention, respectively, treatment center revealed no significant interaction (all interaction *P* ≥ 0.15; Table 3).

**Table 3:** Models additionally adjusted for glucose metrics, PICU-stay duration and for interaction with center and randomization

Neurodevelopmental outcome after 4 years^1^	Working memory		Planning and organization		Metacognition	
	β-Estimate (95% CI)	P	β-Estimate (95% CI)	P	β-Estimate (95% CI)	P
**Model adjusted for baseline risk factors, glucose metrics and length of stay in PICU (model 1)**						
Hypoglycemia	1.98 (0.39 to 3.57)	0.015	1.73 (0.21 to 3.24)	0.026	1.60 (0.01 to 3.19)	0.049
*After additional adjustment for baseline NSE/S100B*						
Hypoglycemia	2.13 (0.43 to 3.83)	0.014	1.87 (0.24 to 3.51)	0.025	1.74 (0.04 to 3.45)	0.045
**Model 1, additionally adjusted for the interaction of hypoglycemia with center**						
Hypoglycemia	3.04 (0.78 to 5.31)	0.009	2.21 (0.05 to 4.38)	0.045	2.62 (0.36 to 4.88)	0.023
Hypoglycemia*center (Leuven)	− 1.49 (− 3.74 to 0.77)	0.20	− 0.68 (− 2.83 to 1.47)	0.54	− 1.42 (− 3.67 to 0.83)	0.21
*After additional adjustment for baseline NSE/S100B*						
Hypoglycemia	3.99 (1.19 to 6.79)	0.005	3.19 (0.49 to 5.89)	0.021	3.61 (0.80 to 6.42)	0.012
Hypoglycemia*center (Leuven)	− 2.30 (− 5.04 to 0.45)	0.10	− 1.62 (− 4.27 to 1.02)	0.23	− 2.30 (− 5.06 to 0.45)	0.10
**Model 1, additionally adjusted for the interaction of hypoglycemia with randomization**						
Hypoglycemia	2.06 (0.38 to 3.74)	0.016	1.90 (0.30 to 3.50)	0.020	1.82 (0.15 to 3.50)	0.033
Hypoglycemia*randomization (Late-PN)	− 0.25 (− 1.83 to 1.33)	0.75	− 0.53 (− 2.02 to 0.97)	0.49	− 0.68 (− 2.25 to 0.89)	0.40
*After additional adjustment for baseline NSE/S100B*						
Hypoglycemia	2.56 (0.69 to 4.42)	0.007	2.38 (0.59 to 4.18)	0.009	2.40 (0.53 to 4.27)	0.012
Hypoglycemia*randomization (Late-PN)	− 0.97 (− 2.69 to 0.76)	0.27	− 1.14 (− 2.80 to 0.52)	0.18	− 1.47 (− 3.20 to 0.26)	0.096

PICU: pediatric intensive care unit

Baseline risk factors include age, sex, treatment center, randomization group, pediatric index of mortality (PIM3 score) at time of admission, Screening Tool for Risk on Nutritional Status and Growth (STRONGkids) risk level at time of admission, diagnostic category, history of malignancy, predefined syndrome, race, geographic origin, language, parental educational level, parental occupational level, parental smoking before admission, left-hand preference, and (where applicable, as indicated in the table) upon-admission concentrations of NSE and S100B. As no patient had diabetes, diabetes was not included in the multivariable models. All models were adjusted for glucose metrics in PICU (mean morning blood glucose and SD of all glucose measurements in PICU) and for length of stay in PICU

^1^Higher scores reflect worse performance

### Association between hypoglycemia and 4-year neurodevelopmental outcomes after additional adjustment for baseline NSE and S100B

Of 674 patients in the neurodevelopmental cohort, 556 patients (82.5%) had baseline blood samples available (*N* = 501 with admission sample, and *N* = 55 with day 1 sample as surrogate), of whom 56 developed hypoglycemia in PICU. Baseline and clinical characteristics, as well as outcomes of these patients, are shown in Table 1. As in the total cohort, patients with hypoglycemia were significantly younger (*P* < 0.0001), more often treated in Leuven (*P* = 0.0003), more frequently randomized to Late-PN (*P* = 0.004), categorized in different diagnostic groups (*P* = 0.0002), and had a higher baseline illness severity score (*P* < 0.0001). Baseline NSE concentrations were significantly higher in patients with than in patients without hypoglycemia (*P* = 0.001), and baseline S100B concentrations tended to be higher (*P* = 0.078). Patients with hypoglycemia had a lower mean morning blood glucose in PICU (*P* < 0.0001), a trend toward higher glucose variability (*P* = 0.055), and a significantly longer duration of PICU stay (*P* < 0.0001). As in the total neurodevelopmental follow-up cohort, univariate comparison revealed significantly worse scores for working memory (*P* = 0.006), planning and organization (*P* = 0.008), and metacognition (*P* = 0.025). In multivariable analyses adjusted for baseline risk factors including NSE and S100B, hypoglycemia remained independently associated with worse scores on working memory (*P* = 0.006), planning and organization (*P* = 0.006), and metacognition (*P* = 0.016) (Table 2). Further adjustment for mean morning blood glucose, glucose variability and length of PICU stay did not alter these findings (*P* = 0.014 for working memory, *P* = 0.025 for planning and organization, and *P* = 0.045 for metacognition; Table 3). Further adjustment of the models for a potential interaction between hypoglycemia and treatment center or randomization did reveal significant interactions. While hypoglycemia remained significantly associated with the three affected neurodevelopmental outcomes (all *P* < 0.05), patients experiencing hypoglycemia in Leuven were protected against worse scores on working memory and metacognition (both interaction *P* = 0.10), and patients with hypoglycemia randomized to late-PN had less impaired scores on metacognition (interaction *P* = 0.096) (Table 3).

### Association between iatrogenic versus spontaneous/recurrent hypoglycemia and outcomes

Of 60 patients who experienced hypoglycemia in PICU and had 4-years neurodevelopmental follow-up, 32 had an episode of iatrogenic hypoglycemia (9 randomized to Early-PN, 23 randomized to Late-PN), whereas 28 patients developed spontaneous/recurrent episodes of hypoglycemia (11 randomized to Early-PN, 17 randomized to Late-PN). In univariable analyses, there was a significant difference in scores for working memory (*P* = 0.008) and planning and organization (*P* = 0.032) across the three cohorts (patients without hypoglycemia, patients with iatrogenic hypoglycemia, and patients with spontaneous/recurrent hypoglycemia) (Table 4). Patients developing spontaneous/recurrent hypoglycemia had worse scores on working memory (*P* = 0.002) and planning and organization (*P* = 0.023) than patients without hypoglycemia, and a significantly worse score for working memory (*P* = 0.043) than patients with an iatrogenic hypoglycemic event. In contrast, patients with an episode of iatrogenic hypoglycemia had no significantly different scores as compared with patients without hypoglycemia (all *P* > 0.15), although the score for planning and organization was also not significantly different from patients with spontaneous/recurrent hypoglycemia (*P* = 0.32) (Table 4). After adjustment for potential confounders, categorization into the three cohorts remained significantly associated with worse scores on working memory (*P* = 0.012), whereby patients with spontaneous/recurrent hypoglycemia had significantly worse scores than patients without hypoglycemia (*P* = 0.003) and a trend toward worse scores than patients with iatrogenic hypoglycemia (*P* = 0.090). Patients with an iatrogenic hypoglycemic event did not perform worse on this test than patients without hypoglycemia (*P* = 0.45) (Table 4).

**Table 4:** Association of iatrogenic versus spontaneous hypoglycemia with outcome, as compared to no hypoglycemia

	Working memory	Planning and organization	Metacognition
T-Score, mean (SD)^1^			
No hypoglycemia	51.4 (11.1)	49.9 (10.2)	50.1 (10.9)
Iatrogenic hypoglycemia	52.8 (11.9)	52.4 (10.6)	52.3 (11.6)
Spontaneous or recurrent hypoglycemia	59.1 (13.2)	56.3 (14.8)	56.0 (14.9)
Univariable comparison, *P*-value			
Overall	0.008	0.032	0.092
Spontaneous vs. no hypoglycemia	0.002	0.023	0.042
Iatrogenic vs. no hypoglycemia	0.59	0.16	0.37
Spontaneous vs. iatrogenic hypoglycemia	0.043	0.32	0.32
Multivariable *P*-value^2^			
Overall	0.012	0.052	0.094
Spontaneous vs. no hypoglycemia	0.003	0.022	0.040
Iatrogenic vs. no hypoglycemia	0.45	0.29	0.36
Spontaneous vs. iatrogenic hypoglycemia	0.090	0.33	0.36

^1^Higher scores reflect worse performance

^2^Multivariable linear regression analyses are adjusted for age, sex, treatment center, randomization group, pediatric index of mortality (PIM3 score) at time of admission, Screening Tool for Risk on Nutritional Status and Growth (STRONGkids) risk level at time of admission, diagnostic category, history of malignancy, predefined syndrome, race, geographic origin, language, parental educational level, parental occupational level, parental smoking before admission, left-hand preference, mean morning blood glucose, SD of all glucose measurements in PICU, and duration of PICU stay. As no patient had diabetes, diabetes was not included in the multivariable model

## Discussion

In this secondary analysis of the PEPaNIC RCT, critically ill children who developed hypoglycemia in PICU had a higher short- and long-term mortality risk as well as worse scores on working memory, planning and organization, and metacognition after 4 years as compared with patients without hypoglycemia in univariable analyses. Patients with hypoglycemia were younger, had a higher baseline illness severity and higher baseline NSE concentrations, and were more likely to be randomized to Late-PN and to receive tight glucose control in Leuven. PICU stay was longer in patients with hypoglycemia, with lower mean morning blood glucose and larger glucose variability. Yet, also after adjustment for these potential confounders, hypoglycemia remained independently associated with poorer executive functions after 4 years, but not with increased mortality. The association of hypoglycemia with poorer executive functions revealed a potential interaction with the randomized nutritional strategy and the treatment center, whereby patients experiencing hypoglycemia under tight glucose control in Leuven and patients developing hypoglycemia while randomized to Late-PN had less impacted scores. In sensitivity analyses, especially spontaneous/recurrent hypoglycemia was independently associated with worse scores for the affected neurodevelopmental outcomes.

Prolonged, severe hypoglycemia may induce neuronal damage and death [3]. However, it remains debated whether a brief episode of iatrogenic hypoglycemia by itself induces harm in critically ill children, since the occurrence of hypoglycemia closely associates with illness severity, with sicker patients and patients with prolonged PICU stay having an increased risk of developing hypoglycemia [3, 9, 24], as confirmed by the current study. For evident reasons, the potential harm of hypoglycemia can only be investigated in observational studies that carry an inherent risk of residual confounding. In a nested case–control study, our group has shown previously that children with hypoglycemia already had higher NSE and S100B concentrations upon PICU admission, levels that did not increase after a hypoglycemic event [9]. Moreover, 4-year neurodevelopmental follow-up of patients randomized to tight versus liberal blood glucose control did not reveal long-term harm from a single episode of iatrogenic hypoglycemia [8]. In contrast, 1-year follow-up of children who had congenital heart surgery included in a RCT on tight glucose control suggested poorer neurodevelopmental outcome in children experiencing hypoglycemia, although this difference was no longer significant at 3-year follow-up [25, 26]. However, in the latter RCT, only 8 patients with hypoglycemia had neurodevelopmental follow-up at 1 year (6 after 3 years), and several important confounders, including ICU length of stay and baseline NSE and S100B, were not taken into account [25, 26]. The current study confirms a significant association of hypoglycemia with some impaired neurodevelopmental outcomes after 4 years in a much larger dataset, also after adjusting for potential confounders including mean glucose, glucose variability, the duration of PICU stay, and baseline NSE and S100B.

The results contrast with our previous 4-year neurodevelopmental follow-up study of critically ill children randomized to tight versus liberal glucose control showing no harm by hypoglycemia [8], which may be explained by the different context. Indeed, the previous study was performed in a context of early PN, in which most episodes of hypoglycemia were insulin-induced [7, 8]. In the current study, the majority of patients developing hypoglycemia did not receive early PN, and participating centers had varying glucose control protocols, going from tight over intermediate to liberal glucose control. Approximately half of the patients with hypoglycemia had spontaneous/recurrent hypoglycemia, and we found that the independent association of hypoglycemia with poorer executive functions was most pronounced in patients with spontaneous/recurrent hypoglycemia. This may indicate that spontaneous/recurrent hypoglycemic events are indicators of the underlying illness severity and the higher intrinsic risk of poor neurodevelopmental outcome, and that a single episode of iatrogenic hypoglycemia may not induce additional harm [5, 8, 27]. This may also explain why there was a significant interaction with center for working memory and metacognition, whereby patients developing hypoglycemia under tight glucose control in Leuven had less affected test results. However, there was no such interaction for planning and organization, and we may lack power to detect a less pronounced impairment in neurodevelopmental outcome in patients with iatrogenic hypoglycemia.

Theoretically, harm by hypoglycemia may depend on the duration of the event, the metabolic rate, and the availability of alternative energy substrates. In this regard, ketone bodies can serve as alternative energy substrate during hypoglycemia [28]. We have previously shown that withholding early PN induces ketogenesis in critically ill children, which statistically mediated part of the outcome benefit of the intervention [12]. In the current study, we found a potential interaction with the randomized nutritional strategy, revealing that patients who developed hypoglycemia under Late-PN had less impacted metacognition scores. However, a considerable number of hypoglycemic events in patients randomized to Late-PN were insulin-induced, and insulin is a strong suppressor of ketogenesis [29]. It remains unclear whether circulating ketones are suppressed in all cases of iatrogenic hypoglycemia, also when induced by low doses of insulin as is expected for Late-PN patients [10]. Unfortunately, we did not have stored samples taken at the time of hypoglycemia to study a potential protective role of ketone bodies.

Outside critical illness, the association of hypoglycemia with long-term neurodevelopmental outcome in children has been studied, especially in neonates who may develop transitional hypoglycemia [30]. Whereas some studies did show association between neonatal hypoglycemia and long-term neurocognitive impairment [30], other studies did not and raised concern regarding potential harm by overzealous glucose administration to treat hypoglycemia [31, 32]. Potential harm associated with hypoglycemia may also be transient. In a large prospective cohort study that investigated at-risk neonates who were screened and treated to maintain a blood glucose of at least 47 mg/dL (2.6 mmol/L), children with neonatal hypoglycemia did not differ with regard to neurological development at 2 years and educational performance at 9–10 years [33, 34], whereas there was an increased risk of poor executive function at 4.5 years [35]. In the current study, a considerable proportion of patients with hypoglycemia were infants at time of inclusion and hence they were followed up at an age between 4 and 5 years. It remains unclear whether potential harm associated with hypoglycemia at this follow-up moment will translate into persistent impairment in childhood and adulthood. Moreover, the vulnerability to hypoglycemia and exact blood glucose threshold associated with long-term harm after PICU stay may be age-dependent [36], a possibility that requires further study. In the current study, children who developed hypoglycemia were younger than patients who did not develop hypoglycemia. Although this may suggest that the youngest children are more prone to develop hypoglycemia, it may also be explained by age-dependent targets for blood glucose control. Indeed, hypoglycemia more frequently occurred in Leuven, where the blood glucose target range was lower for infants than for older children.

The current study results will likely fuel the debate on the ideal blood glucose target for critically ill children. Although both hyperglycemia and hypoglycemia are associated with poor outcome in critically ill children [1, 5], RCTs have not shown consistent benefit of tight glucose control [7, 37–40]. Whereas a single-center RCT in critically ill children receiving early PN has found improved morbidity and mortality by treating hyperglycemia with insulin [7], subsequent multicenter RCTs have been neutral [37, 38, 40]. Although the absence of benefit in the pediatric multicenter RCTs may be explained by a very small difference in blood glucose concentration between study groups and consequently, lack of statistical power [41, 42], tight glucose control inherently increases the risk of hypoglycemia, which could counteract any potential benefit. Mechanistic studies have attributed the benefit of tight glucose control with intensive insulin therapy to avoidance of glucose overload in vital organs rather than to glycemia-independent effects of insulin [43]. The current feeding practice of withholding PN in the acute phase of critical illness not only lowers the severity of hyperglycemia, it also increases the risk of hypoglycemia [10, 44]. Hence, the risk–benefit of preventing less severe hyperglycemia in this context remains to be studied. Also in critically ill adults, the efficacy and safety of tight glucose control in the absence of early PN is debated, which is currently being studied in the TGC-fast multicenter RCT [45]. Regardless of the results of this RCT and the blood glucose target aimed for, any glucose control protocol should minimize the incidence of hypoglycemia by frequent and accurate blood glucose measurements and prompt correction of hypoglycemia, while avoiding overtreatment.

The study is a secondary analysis of a large multicenter RCT with prospective collection of long-term mortality data in all patients and detailed neurodevelopmental follow-up in a large subset of patients, which is a strength. However, the study also has limitations. The study is observational, and we did not correct for multiple comparisons, because the studied neurodevelopmental outcomes are not independent [11]. Hence, we cannot exclude residual confounding. Moreover, executive functions were reported by parents or caregivers. However, in view of the consistent signal for harm, it seems cautious to consider this signal as potentially clinically relevant. Second, the association with long-term neurodevelopmental outcome could only be investigated in the subset of patients with available data and after exclusion of patients in Edmonton, since none of the three children developing hypoglycemia in Edmonton had neurodevelopmental follow-up. However, the association with short- and long-term mortality was investigated in all study patients. Third, the sensitivity analyses regarding impact of spontaneous/recurrent hypoglycemia versus single event of iatrogenic hypoglycemia were done in relatively smaller cohorts, which may have reduced the statistical power to detect any difference. Fourth, we used treatment center as proxy for the center-specific glucose control protocol. A RCT would be needed to directly study the impact of different glucose control strategies on the association of hypoglycemia with outcome. Fifth, in the absence of continuous glucose monitoring, episodes of hypoglycemia can be missed and information on the exact duration of a hypoglycemic episode is lacking. However, intermittent rather than continuous glucose monitoring is likely reflective of routine clinical practice in most centers. Finally, we could not investigate whether diabetes patients may be more vulnerable to hypoglycemic damage [46], in view of the very small number of critically ill children with diabetes.

## Conclusion

Critically ill children who experienced hypoglycemia during PICU stay, mostly those with spontaneous/recurrent hypoglycemia rather than a single event of iatrogenic hypoglycemia, were found to be at higher risk of developing impaired executive functions 4 years later, also when adjusted for risk factors.

## Data Availability

Data sharing is offered under the format of collaborative projects. Proposals can be directed to the corresponding author.

## Abbreviations

BRIEF: Behavior Rating Inventory of Executive Function
CBCL: Child Behavior Checklist
ELISA: Enzyme-linked immunosorbent assay
NSE: Neuron-specific enolase
PICU: Pediatric intensive care unit
PIM3: Pediatric index of mortality 3
PN: Parenteral nutrition
RCT: Randomized controlled trial
STRONGkids: Screening Tool for Risk On Nutritional Status and Growth
S100B: S100 calcium binding protein B
